# *Bacillus velezensis* AP183 Inhibits *Staphylococcus aureus* Biofilm Formation and Proliferation in Murine and Bovine Disease Models

**DOI:** 10.3389/fmicb.2021.746410

**Published:** 2021-10-08

**Authors:** Sayma Afroj, Andrew D. Brannen, Shamima Nasrin, Abdulaziz Al Mouslem, Terri Hathcock, Herris Maxwell, Cody R. Rasmussen-Ivey, Mary J. Sandage, Edward W. Davis, Peter Panizzi, Chengming Wang, Mark R. Liles

**Affiliations:** ^1^Department of Biological Sciences, Auburn University, Auburn, AL, United States; ^2^Department of Drug Discovery and Development, Auburn University, Auburn, AL, United States; ^3^Department of Pathobiology, Auburn University, Auburn, AL, United States; ^4^Department of Clinical Sciences, Auburn University, Auburn, AL, United States; ^5^Department of Speech, Language, and Hearing Sciences, Auburn University, Auburn, AL, United States; ^6^Department of Mechanical Engineering, Auburn University, Auburn, AL, United States

**Keywords:** *Staphylococcus aureus*, *Bacillus velezensis*, biocontrol, infection, cutaneous, mastitis, biofilm, tracheostomy tube

## Abstract

The increasing frequency of *S. aureus* antimicrobial resistance has spurred interest in identifying alternative therapeutants. We investigated the *S. aureus-*inhibitory capacity of *B. velezensis* strains in mouse and bovine models. Among multiple *B. velezensis* strains that inhibited *S. aureus* growth *in vitro*, *B. velezensis* AP183 provided the most potent inhibition of *S. aureus* proliferation and bioluminescence in a mouse cutaneous wound (*P* = 0.02). Histology revealed abundant Gram-positive cocci in control wounds that were reduced in *B. velezensis* AP183-treated tissues. Experiments were then conducted to evaluate the ability of *B. velezensis* AP183 to prevent *S. aureus* biofilm formation on a tracheostomy tube substrate. *B. velezensis* AP183 could form a biofilm on a tracheostomy tube inner cannula substrate, and that this biofilm was antagonistic to *S. aureus* colonization. *B. velezensis* AP183 was also observed to inhibit the growth of *S. aureus* isolates originated from bovine mastitis cases. To evaluate the inflammatory response of mammary tissue to intramammary inoculation with *B. velezensis* AP183, we used high dose and low dose inocula in dairy cows. At the high dose, a significant increase in somatic cell count (SCC) and clinical mastitis was observed at all post-inoculation time points (*P* < 0.01), which resolved quickly compared to *S. aureus-*induced mastitis; in contrast, the lower dose of *B. velezensis* AP183 resulted in a slight increase of SCC and no clinical mastitis. In a subsequent experiment, all mammary quarters in four cows were induced to have grade 1 clinical mastitis by intramammary inoculation of a *S. aureus* mastitis isolate; following mastitis induction, eight quarters were treated with *B. velezensis* AP183 and milk samples were collected from pretreatment and post-treatment samples for 9 days. In groups treated with *B. velezensis* AP183, SCC and abundance of *S. aureus* decreased with significant reductions in *S. aureus* after 3 days post-inoculation with AP183 (*P* = 0.04). A milk microbiome analysis revealed significant reductions in *S. aureus* relative abundance in the AP183-treated group by 8 days post-inoculation (*P* = 0.02). These data indicate that *B. velezensis* AP183 can inhibit *S. aureus* biofilm formation and its proliferation in murine and bovine disease models.

## Introduction

*S. aureus* is commonly responsible for cutaneous infections in human and veterinary medicine, with the rising prevalence of antibiotic resistant strains prompting the need for novel strategies to combat infection (Guo et al., 2020). Damage to the integument from injury, including surgical implants such as tracheostomy tubes (El Cheikh et al., 2018), creates an opportunity for opportunistic pathogens like *S. aureus* to colonize and often precedes systemic infection (Ki and Rotstein, 2008). Asymptomatic carriage of *S. aureus* in humans is estimated to be approximately 20 percent, though the pathogen is common to a variety of acute and chronic skin pathologies including impetigo, cellulitis, furuncles, scalded skin syndrome, and mastitis (Iwatsuki et al., 2006; Delgado et al., 2011). Cutaneous infections are burdensome in agriculture as well, with bovine mastitis alone resulting in an estimated annual cost exceeding $1.7 billion to the dairy industry and *S. aureus* as the causative pathogen in 20% of cases (Wells and Ott, 1998; Shim et al., 2004). Intramammary infection (IMI) resulting in mastitis poses significant financial and production losses in dairy cows and other dairy species, including decreased milk production, increased culling rates, and increased production costs due to treatment (Halasa et al., 2007; Mekibib et al., 2010; Heikkila et al., 2012; Leslie and Petersson-Wolfe, 2012; Ruegg, 2017). The primary causative agents of IMI are bacterial, including *Staphylococcus aureus*, *Staphylococcus epidermidis*, *Streptococcus agalactiae*, *Streptococcus uberis*, and *Escherichia coli* (Barkema et al., 2009; Piessens et al., 2011; Supre et al., 2011). Coagulase-negative staphylococci are frequently associated with IMI (Piessens et al., 2011) while coagulase-positive *Staphylococcus* spp., such as *S. aureus*, can induce acute and chronic IMI that are often difficult to control (Roberson et al., 1994; Almeida et al., 1996; Contreras and Rodriguez, 2011; Bouchard et al., 2013). Antibiotic use remains the most common treatment against mastitis (Barlow, 2011); however, the use of antibiotics may not only foster bacterial antimicrobial resistance, but can also cause contamination of milk and meat, potentially contributing to dissemination of antibiotic resistance in the environment and in humans (Oliver and Murinda, 2012; Sharma et al., 2017).

The frequency of antibiotic resistance among *S. aureus* strains is of particular concern and may be the result of the acquisition of mobile genetic elements encoding antibiotic resistance (Davis et al., 2016). More than 80,000 severe human infections are caused every year in the United States from methicillin-resistant *S. aureus* (MRSA) (Solomon and Oliver, 2014). Worldwide, this has become the most common infection due to drug-resistant pathogens, with reservoirs of drug-resistant *S. aureus* within hospitals and in livestock (Lakhundi and Zhang, 2018). Mounting concerns have recently led to updated guidance for antibiotic use in livestock by the Food and Drug Administration, including a federal government initiative to promote voluntary elimination of antibiotics in livestock for growth purposes (Greer, 2016). The rapidly growing threat of antibiotic resistant pathogens in humans and the impending restrictions on antibiotic use in livestock illustrate the need for innovative strategies for controlling the spread of antibiotic-resistant pathogens.

Probiotics may be an effective alternative to antibiotics to prevent or treat *S. aureus* infections. For example, using probiotic treatment against *S. aureus* infections includes the ability of the probiotic to effect pathogen inhibition by multiple mechanisms, including competitive exclusion (Hojjati et al., 2020), direct antagonism via metabolites (Mohamed et al., 2020), and/or stimulation of the host immune response (Liu et al., 2020). The multifactorial action of probiotics has also been shown to preclude the development of pathogen resistance; for example, studies of intramammary infusion with different *Lactobacillus* spp. (*L. lactis*, *L. perolens*, *L. rhamnosus*, *L. brevis*, and *L. plantarum*) were shown to be effective agents in the prevention and treatment of mastitis (Frola et al., 2012; Bouchard et al., 2013; Gomes and Henriques, 2016; Camperio et al., 2017; Mignacca et al., 2017). Other results indicate that *Lactobacillus* strains are not effective in decreasing bovine mastitis and instead initiate an inflammatory response (Frola et al., 2013). So far, few studies have assessed the *in vivo* effects of probiotics in the mammary gland. Catozzi et al. (2019) investigated intramammary treatment with *L. rhamnosus* in water buffalo and observed transient pro-inflammatory activity and modification of the milk microbiota. Most of these studies did not evaluate efficacy in chronic clinical mastitis cases. Moreover, the inhibitory activities of these previously evaluated probiotic strains were not observed to be highly potent against major mastitis pathogens such as *S. aureus*. To develop an effective probiotic strategy against mastitis, identification of novel probiotics with potent antagonistic activity is crucial, and there is a benefit in using spore-forming species in the *B. subtilis* group, including the species *B. amyloliquefaciens* and *B. velezensis*, that have a long shelf-life, produce many bioactive metabolites and are known to be safely used in agriculture and aquaculture (Kloepper et al., 1980; Cook et al., 1995; Ongena and Jacques, 2008; Ran et al., 2012; Hossain et al., 2015; Fan et al., 2018; Kaspar et al., 2019; Ngalimat et al., 2021), but few strains have been examined for potential clinical applications (Geeraerts et al., 2015; Wang et al., 2017).

Our previous research screened 177 spore-forming *Bacillus* spp. isolates that were primarily cultivated from plant rhizospheres for the ability to inhibit MRSA growth, from an extensive microbial collection containing thousands of unique isolates cultivated at Auburn University by the research group of Prof. Joseph Kloepper (Hollis, 2020). Among these MRSA-inhibiting isolates, rhizosphere-derived *B. velezensis* AP183 was found to produce bacillusin A, a novel and potent macrodiolide antibiotic capable of inhibiting methicillin-resistant *S. aureus* and vancomycin-resistant *Enterococcus faecium* (Ravu et al., 2015). In addition to bacillusin A, *B. velezensis* AP183 and many of these other *Bacillus* spp. strains are known to produce other metabolites with anti-bacterial (e.g., difficidin, surfactin, diketopiperazine, and macrolactin) or anti-fungal (e.g., iturin) activities (Ravu et al., 2015). Interestingly, bacillusin A was shown to be highly labile, suggesting that the *in vivo* effects of this strain would be localized and transient; however, while each of these *Bacillus* spp. strains produced secondary metabolite(s) under *in vitro* conditions that inhibited MRSA growth, none of these *Bacillus* spp. strains had been evaluated for their ability to prevent *S. aureus* infection under *in vivo* conditions. In this study, we began our investigation by testing five different *Bacillus* spp. strains for their respective ability to prevent *S. aureus* infection in a murine cutaneous wound model, as a first step toward investigation of the use of a specific *Bacillus* spp. strain for biocontrol of *S. aureus* infections in clinical and veterinary medicine.

## Materials and Methods

### Microorganisms and Growth Conditions – *Mouse Experiments*

A commercially available *S. aureus* Xen29 (PerkinElmer Inc., Waltham, MA, United States) was used for *in vivo* cutaneous wound challenge studies (Table 1). *S. aureus* Xen29 from glycerol stocks stored at –80°C were streaked for isolation onto Brain-Heart Infusion (BHI)(Research Products International Corp., Mt. Prospect, IL, United States) agar plates containing 50 mg/mL of kanamycin monosulfate (Research Products International Corp., Mt. Prospect, IL, United States) and incubated for 16 h at 37°C. Following incubation, plates were imaged using a LAS-1000 luminescent image analyzer (FUJIFILM Corporation) to confirm bioluminescence. One colony was selected to inoculate a flask containing 50 mL of BHI containing 50 mg/mL of kanamycin monosulfate. The culture was incubated at 37°C for 16 h, shaking at 225 RPM. The bacterial suspension was subjected to centrifugation at 2,200 × *g* for 15 min at room temp. The supernatant was discarded, and the pellet was resuspended in 20 mL of sterile-filtered 1 × PBS, followed by centrifugation at 2,200 × *g* for 15 min at room temp. The supernatant was again discarded, and the pellet was suspended in 5 mL of sterile-filtered 1 × PBS with 10% glycerol. Serial dilutions of the bacterial suspension were diluted into sterile-filtered PBS with 10% glycerol and the optical density was determined at 600 nm (OD_600_). According to the manufacturer’s instructions, an optical density of 0.5 units is approximately equivalent to 1.4 × 10^8^ CFU/mL.

**TABLE 1:** Bacterial isolates used in this study.

**Organism**	**Strain**	**Source or reference**
*B*. *velezensis*	AP143	This study^1^
*B*. *velezensis*	AP191	This study^1^
*B*. *velezensis*	AP183	This study^1^
*B*. *velezensis*	AP218	This study^1^
*B*. *velezensis*	AB01	This study^1^
*S. aureus*	Xen29	PerkinElmer (Akron, OH, United States)
*S. aureus*	NE1260F	Bose et al., 2013
*S. aureus*	4366-13	This study^2^
*S. aureus*	1171-09	This study^2^
*S. aureus*	1052-1	This study^2^
*S. aureus*	2226-11	This study^2^
*S. aureus*	1779-09	This study^2^
*S. aureus*	3851-07	This study^2^
*S. aureus*	3651-09	This study^2^
*S. aureus*	RF 122	This study^2^
*S. uberis*	22272-08	This study^2^
*K. pneumoniae*	1583-07	This study^2^
*B. cereus*	NRS 1595	This study^2^

*^1^Department of Entomology and Plant Pathology, Auburn University Microbial Culture Collection.*

*^2^College of Veterinary Medicine, Auburn University.*

*Bacillus* spp. strains were cryopreserved in Tryptic Soy Broth (TSB) containing 20% glycerol. Each strain was grown on Tryptic Soy Agar (TSA) overnight at 30°C to obtain isolated colonies. *Bacillus* spp. spores used for *in vivo* challenge were prepared by inoculating an isolated bacterial colony into a 20 mL culture tube containing 5.0 mL of TSB and incubated on a rotatory shaker at 200 RPM at 30°C for 5 days, after which the vegetative cells were killed by heating at 80°C for 20 min, and the CFU/mL were determined by serial dilution and plating onto TSA. To prepare for injection, spores were dispensed into 1.0 mL aliquots with 10% glycerol.

### *In vivo* Cutaneous Wound Model

Experimental protocols were reviewed, approved, and performed under regulatory supervision of Auburn University’s Institutional Biosafety Committee (IBC) and Institutional Animal Care and Use Committee (IACUC), under IACUC protocols 2011-2006 and 2016-2988. For this study, 22 female C57BL/6J mice at 6–8 weeks old were anesthetized by 1–3% isoflurane gas and hair was shaven with electronic clippers and depilated using Veet^®^ (Reckitt Benckiser Group, Berkshire, United Kingdom). Treatment groups (*n* = 3 mice per *Bacillus* strain) were injected subcutaneously between the shoulder blades with a 20 mL suspension comprised of 10 mL 1.0 × 10^8^ CFU/mL *S. aureus* Xen29 and 10 mL of 1 × 10^8^ CFU/mL *B. velezensis* spores with metabolites (total of 1.0 × 10^6^ CFUs) for strains AB01, AP143, AP183, AP191, and AP218. Internal controls were administered by subcutaneous injection of 1.0 × 10^6^ CFUs of *S. aureus* Xen29 in a volume of 10 mL on the lower back. Untreated control mice (*n* = 3) were given subcutaneous injections of 1.0 × 10^6^ CFUs of *S. aureus* Xen29 between the shoulder blades and the lower back. In a subsequent experiment, a group of mice was administered subcutaneous injections of AP183 spores (1 × 10^6^ CFU/mL) with associated metabolites between the shoulder blades and lower back and another group was injected subcutaneously with metabolites only between the shoulder blades and lower back of each animal. At the end point of the experiment at 6 days post-inoculation, all mice were sacrificed by CO_2_ asphyxiation, followed by cervical dislocation, and wound samples were harvested for histological analysis and homogenized for CFU determination.

### *In vivo* Imaging Procedures

All *in vivo* imaging was performed using an IVIS Lumina XRMS (Caliper Life Sciences). Mice were anesthetized by 1–3% isoflurane gas and placed in the imaging chamber of the IVIS Lumina XRMS. Bioluminescence exposures were 5 min with medium binning, *f-2* aperture, and a 12.5 cm × 12.5 cm field of view. All mice were imaged once per day for a period of 7 days. At the endpoint, mice were sacrificed according to IACUC protocol guidelines and wound sites were excised and stored in 1 × PBS. Wound samples were split in half, with one half embedded for histological analysis and the other half homogenized for DNA isolation (see DNA isolation section below) to enable 16S rRNA gene amplicon sequencing and serial dilutions were plated to determine final CFU/mg of tissue.

### Evaluation of AP183 Formulations for *in vivo* Administration

To determine the optimal formulation for administration of AP183 to wound sites, three formulations were prepared and tested *in vivo* for inhibition of *S. aureus* proliferation. AP183 spores (1.0 × 10^6^ CFUs), supernatant containing AP183 metabolites without spores, and AP183 spores with supernatant (1.0 × 10^6^ CFUs) were co-administered with 1.0 × 10^6^ CFUs *S. aureus* Xen29. Mice (*n* = 3 per group) in a volume of 10 mL were imaged for bioluminescence once a day for 6 days. At the endpoint, mice were sacrificed by CO_2_ asphyxiation and cervical dislocation. Wound tissue was resected, homogenized, and diluted followed by plating on TSA and incubated for 18 h at 37°C. *S. aureus* colonies were counted by bioluminescent imaging to determine CFU counts. Plates were incubated for 18 h at 37°C and bioluminescence imaging was performed on each plate to allow counting of luminescent colonies and ultimately determine CFUs present in each wound. The other half of each wound was prepared for histological Gram and hematoxylin and eosin (H&E) staining.

### Tissue Staining and Histological Analysis

Wound tissue from two mice from each treatment or control group was embedded in clear frozen section compound (VWR International, West Chester, PA, United States) medium and fixed by submersing in a metal container with 2-methylbutane (Alfa Aesar, Haverhill, MA, United States) on dry ice/liquid nitrogen. Sectioning was performed using a Microm HM 525 (Thermo Fisher Scientific Inc.) with a section thickness of 0.6 mM per slice. Alternating slices were stained with either the following Gram staining or H&E staining procedures. Microscopy was performed using a Zeiss Axioskop with Plan-NEOFLUAR objective lenses and Nikon Sight DS-Fi2 digital camera.

Gram staining of slides was performed by submersion in crystal violet (EMD Millipore, Billerica, MA, United States) for 15 s, followed by gentle rinsing with tap water, submersion in Gram’s Iodine (EMD Millipore, Billerica, MA, United States) for 15 s, tap water rinse, submersion in tartrazine (EMD Millipore, Billerica, MA, United States) solution for 15 s, and a final tap water rinse. Slides rinsed with 95% alcohol and allowed to dry, then dripped with xylene (EMD Millipore, Billerica, MA, United States) and allowed to dry before a coverslip with Cytoseal^TM^ (Thermo Fisher Scientific Inc.) was placed over each slide.

H&E staining was performed by submersing slides in hematoxylin (EMD Millipore, Billerica, MA, United States) and placed on a rocker for 15 min, then rinsed in tap water with gentle shaking for 15 min. Slides were submersed in 95% alcohol for 30 s and submersed into eosin (VWR International, West Chester, PA, United States) for 45 s. Slides were then submerged into 95% alcohol for 1 min, followed by submersion in 100% alcohol for 1 min. Slides were then submersed in xylene for 1 min and allowed to air dry. A coverslip with Cytoseal^TM^ (Thermo Fisher Scientific Inc.) was then placed over each slide prior to microscopic examination.

### Microorganisms and Growth Conditions – *Biofilm Experiment*

A single colony of *B. velezensis* AP183 was inoculated into 3 mL of TSB and cultivated overnight at 37°C with shaking at 250 rpm. Small (∼3 mm) sections of a Shiley^TM^ tracheostomy tube inner cannula (Medtronic, Minneapolis, MN, United States) were sterilized in 70% ethanol and dried prior to placing into a culture tube containing 3 mL of TSB. Then 100 μL of the overnight culture of *B. velezensis* AP183 was added to the tube and incubated for 72 h at 37°C. The supernatant was then removed, and planktonic cells were removed by washing the inner cannula sections three times in 1 × phosphate-buffered saline. After washing the inner cannula sections, 100 μL of an overnight TSB culture of *S. aureus* NE1260F (Bose et al., 2013) was added to each tube and incubated at 37°C with shaking at 250 RPM for 24 h. The experiment was conducted in triplicate, and a negative control was included that only had 100 μL of *S. aureus* culture added to the inner cannula sections, which were incubated at 37°C with shaking at 250 RPM for 24 h. The next day each inner cannula section was washed three times in 1 × phosphate-buffered saline and DNA was extracted from each section (see DNA isolation section below).

### Evaluation of Carbon Sources on *Bacillus velezensis* AP183 Inhibition of MRSA Colonization

In this experiment the competition between *B. velezensis* AP183 and *S. aureus* was evaluated when log-phase cultures were co-inoculated in a 96-well plate containing a defined M9 salts minimal medium containing different carbon sources. Two of the carbon sources (glucose and sucrose) could be utilized for growth by both cultures, whereas pectin could only be utilized by *B. velezensis* AP183 (Hassan et al., 2019). In this experiment a thin film of the carbon source being evaluated (pectin, glucose, or sucrose) was prepared in 96 well plates by evaporative drying from a saturated solution. Each solution was prepared by mixing the respective carbon source with sterilized DI water for 24 h under magnetic stirring. The required amount of solution was then pipetted into the plate wells and the plate dried at 30°C for 24 h. The amount of carbon source added ranged from 1 to 20% (w/v) with four replicates per each concentration. *B. velezensis* AP183 and *S. aureus* NE1260F were inoculated into separate 2 mL TSB cultures and incubated overnight at 37°C with shaking at 250 rpm. The cultures were normalized to OD_600_ = 1.0 and then 1 mL was removed, subjected to centrifugation at 10,000 × *g* and resuspended in 1 mL of M9 minimal salts medium. The *B. velezensis* AP183 and *S. aureus* NE1260F cells were then diluted 1:100 into the same tube of M9 medium, and 20 mL of this stock was added to each well of the 96-well plate containing different carbon sources (for a total 1:1000 dilution, approximately 10^6^ CFU of each culture in each well) with a total volume of 200 mL per well. The 96-well plate was incubated for 72 h at 37°C with shaking at 250 rpm and then each well was serially diluted and plated on TSA to determine CFU/mL for each of the two cultures, which were discriminated based on their unique colony morphologies. Due to the expected inhibition of *S. aureus* viability by *B. velezensis* AP183 when grown on TSA at high densities, only plates with lower numbers of CFUs (<100) were selected for calculation of CFU/mL.

### Microorganisms and Growth Conditions – *Dairy Experiments*

The bacterial strains used in this study included the mastitis isolate *S. aureus* RF122 and other bacterial isolates isolated from clinical mastitis cases (Table 1). All *Bacillus* and *Staphylococcus* cultures were cryopreserved in TSB containing 20% glycerol and isolated colonies obtained on TSA. The antimicrobial activity of *B. velezensis* AP183 against bacteria isolated from mastitis cases (Table 1) was assayed using a soft agar overlay technique. The assay was performed in triplicate and inhibitory activity was quantified by measuring the zone of inhibition in mm.

To generate a *S. aureus* RF122 culture for intramammary inoculation, a single colony was inoculated into 3 mL of TSB and cultivated at 37°C for 16 h followed by transfer of 1 mL of the bacterial culture to 100 mL of TSB and incubated overnight at 37°C with shaking. After overnight incubation, the culture was normalized to an OD_600_ of 0.5 and then diluted to an approximate concentration of 10^2^ CFU/mL and 10 mL of this diluted culture was used for the intramammary inoculation of bovine quarters.

For cultivation of *B. velezensis* AP183, an isolated colony was inoculated into 3 mL of TSB and grown at 30°C for 18 h followed by transferring 1 mL of the bacterial culture to 100 mL of TSB. After overnight incubation at 30°C with shaking, the final approximate concentration of the bacterial suspension was 10^8^ CFU/mL and 10 mL of this dose was used directly for the high dose *B. velezensis* AP183 treatment containing 10^9^ CFU total. For the low dose treatment, the overnight culture was diluted to 10^2^ CFU/mL and 10 ml was used for intramammary inoculation that contained 10^3^ CFU total. To produce spores of *B. velezensis* AP183, a TSB culture was used to inoculate 10 spore preparation agar plates using a cotton swab and incubated for 10 days at 30°C. The spores were harvested, and the spore suspension was heat treated at 80°C for 20 min to kill vegetative cells. The concentration of the spore suspension was determined by serially diluting the spore suspension in sterile water and incubating in TSA plates for overnight at 30°C. The spore suspension was diluted to 10^4^ CFU/mL and were preserved at 4°C until further use. On the experimental day (D_0_), the final concentration of the spore suspension was adjusted to 10^2^ CFU/mL with sterile water and used for intramammary inoculation.

### Experimental Design for Intramammary Challenge and Sample Collection

The experimental protocol was approved by Auburn University Institutional Animal Care & Use Committee (IACUC Protocol Number: 2017-3120). This study was conducted on four first-lactation Holstein dairy cows, age 2 years, at the Auburn University College of Veterinary Medicine. Animal health status was monitored clinically, and milk quality was monitored by bacteriological culture and somatic cell count analysis of individual quarter milk samples.

Prior to evaluating interactions between *B. velezensis* AP183 and *S. aureus* within mammary tissue, experiments were conducted to assess the impact of *B. velezensis* AP183 alone on mammary inflammatory responses.

In the first experiment a high dose inoculum of *B. velezensis* AP183 (10 mL of a 10^8^ CFU/mL bacterial suspension) was used for an intramammary inoculation in four animals, with half of the 16 healthy mammary glands (a fore and a rear quarter from each cow) inoculated and the other uninoculated quarters considered as negative controls. Surface cleaning was performed with a dry paper towel and a commercial teat dip containing 1% povidone iodine disinfectant for 30 s, followed by disinfecting the teat ends using isopropyl alcohol swabs, milk samples were collected every day from 1 day prior to inoculation (D_–1_), inoculation day (D_0_) and at the post inoculation days from one to fifteen (i.e., D_1_ to D_15_) in 15 mL conical tubes. Milk samples were immediately placed on ice and then transferred to the laboratory for analysis of microbiological counts on TSA, SCC, fat, protein, lactose, and milk solids-not-fat (SNF) content.

In a second experiment, a lower dose inoculum of *Bacillus* AP183 (10 mL of a 10^2^ CFU/mL) was administered intramammary in six healthy bovines, as described above. Milk samples were collected on inoculation day (D_0_) and at the post inoculation days from D_1_ to D_4_, for determination of *Bacillus* CFU counts, SCC, and other raw components. The procedure for quarters disinfection, milk collection, transportation and analysis were the same as described above for the higher dose study.

To investigate the impact of *B. velezensis* AP183 on the microbiology and mammary inflammatory response during *S. aureus*-induced clinical mastitis, 10 mL of a 10^2^ CFU/mL *S. aureus* RF122 culture were administered intramammary in all quarters of four animals, 1 day prior to low dose *B. velezensis* inoculation (D_–__1_). At time D_0_, half of the quarters were subjected to intramammary inoculation with 10 mL of a 10^2^ CFU/mL *B. velezensis* AP183 suspension. Milk samples were collected every day starting the day prior to *S. aureus* inoculation (D_–__2_) and continuing every day to 9 days post-inoculation (i.e., D_9_). The sample handling protocol was the same procedure described above for milk sample collection, transport and analysis. In addition, the milk samples collected from this *S. aureus*-induced mastitis experiment were aliquoted into microcentrifuge tubes and stored at -80^*o*^C until processed for microbiome analysis for samples D_0_, D_2_, D_5_, and D_8_.

### LC-MS Analysis of Milk Samples

Milk samples from the high dose *B. velezensis* AP183 study were analyzed for bacillusin A using liquid chromatography-mass spectrometry (LC-MS) at the Auburn University Mass Spectrometry Lab, using purified bacillusin A diluted into milk known to be without exposure to the macrodiolide. The milk samples and standards were mixed with ice cold acetone (100 μL milk and 400 μL acetone), vortexed for 30 s, frozen for 15 min, subjected to centrifugation at 10,000 × *g* for 5 min, and decanted. Exposure to light was minimized during all sample and standard handling steps. The liquid was evaporated for 4 h in a Thermo Savant DNA 120 speed vac concentrator without any heating. The milk standards and samples were reconstituted in 50 μL of 70% water and 30% acetonitrile, vortexed, and incubated for 5 min in a water bath ultrasonicator, and subjected to centrifugation at 10,000 × *g* for 5 min. The supernatant was analyzed using an ultra-performance LC system (ACQUITY, Waters Corp., United States) coupled with a quadrupole time-of-flight mass spectrometer (Q-Tof Premier, Waters) with electrospray ionization (ESI) in negative mode using Masslynx software (V4.1). Injection of 5 mL of the sample or standard was made on a C18 column (ACQUITY UPLC^®^ BEH C18, 1.7 μm, 1 × 50 mm, Waters) with a 200 μL/min flow rate of mobile phase solution A (0.1% formic acid in 95% water and 5% acetonitrile) and solution B (0.1% formic acid in 95% acetonitrile and 5% H_2_O) beginning at 35% B, held for 1 min followed by a linear ramp to 50% B in 9 min, then to 100% B at 11 min, held 2 min, and back to 35%B with 6 min of re-equilibration. The MS spectral range was 120–1300 m/z with a scan time of 0.1 s and 0.05 interscan delay. The 597.3 ion was chosen for MSMS with maximum sensitivity, collision energy of 30 eV, LM resolution of 4.7, and 0.3 s scan and 0.05 s interscan delay. The capillary voltage was set at 2.8 kV, the sample cone voltage was 30 V, and the extraction cone was 4.0 V. The source and desolvation temperature were maintained at 95 and 400°C, respectively, with the desolvation gas flow at 600 L/h. The lock mass was used to correct instrument accuracy with a 0.1 μM solution of HP 1221 (Agilent part number G1969-85003).

### Dairy Cow Clinical Observations

Cows were pastured with the Auburn University Large Animal Teaching Hospital dairy herd and fed the same ration as the remainder of the herd. Milking was carried out twice daily in a milking parlor. The California mastitis test was performed on each quarter at the time of morning milking and milk was collected for laboratory submission to quantify somatic cell counts, percent lactose, percent protein, percent fat, and percent SNF and bacterial culture. A daily physical examination was conducted immediately following milking each morning with the cows restrained in self-locking stanchions in the feeding area.

### Bacterial and Somatic Cell Count Counting and Raw Milk Component Analysis

Milk samples were analyzed for the determination of SCC by flow cytometry utilizing a Somacount FCM (Bentley Instruments Chaska, MN, United States) and other milk components including lactose, protein, fat, SNF were analyzed using a FTS Fourier Transform Spectrometer (Bentley Instruments) at the Mid-South Dairy Records Laboratory (Springfield, MO, United States). Microbiological enumeration was performed by culture plate count method by serial dilution and plating for CFUs on TSA as described above.

### Genomic DNA Isolation from Wound Homogenates, Biofilm Samples and Milk Samples

Following endpoint procedures, wounds for each respective treatment or control group were excised using a sterile scalpel for homogenization and extraction of genomic DNA as described previously (Nasrin, 2015). In the case of biofilm formed on inner cannula sections, the sections were removed sterilely and placed into DNA isolation tubes. Genomic DNA was extracted from wound homogenates and biofilm samples using an ultraclean microbial DNA isolation kit following the manufacturer’s instruction (MO BIO laboratories Inc., Carlsbad, CA United States). For milk samples, genomic DNA was isolated using a milk bacterial DNA isolation kit (Norgen Biotek, Thorold, ON, Canada). Briefly, the milk samples were subjected to centrifugation at 20,000 × *g* for 2 min using a Microfuge 22R (Beckman Coulter Life Sciences, Indianapolis, IN, United States). The pellet was resuspended and used for DNA isolation according to the manufacturer’s protocol. The purity of the DNA from each sample was determined using a TECAN infinite M1000 PRO and the DNA samples were quantified using Qubit dsDNA HS Assay kit and read with a Qubit 2.0 fluorometer (Invitrogen, Life Technologies) according to manufacturer’s instructions and stored at –20°C until further use.

### Bacterial Relative Abundance Determination by 16S rRNA Gene Amplicon Sequencing

Each genomic DNA sample was used as a template for 16S rRNA gene sequencing using the “universal bacteria” 16S rRNA gene bacterial primer set 515F/926R that targets the hypervariable V4 and V5 regions and a high-fidelity polymerase to generate amplicons for sequencing using a MiSeq sequencer (Illumina, San Diego, CA, United States), with a 2 × 300 paired-end v2 sequencing kit and a 30% phiX control, at the University of Illinois at Chicago’s Sequencing Core. Changes in the relative abundance of bacterial taxa were assessed using the Python-based QIIME bioinformatics pipeline v.1.9.1 at the family and genera phylogenetic levels (Kuczynski et al., 2012). The specific order of processing was as follows: library generation (files available upon request), barcode trimming with the script split_libraries.py, selection of operational taxonomic units (OTUs) with the script pick_otus.py, selection of a representative set with the script pick_rep_set.py, assignment of taxonomy with the script assign_taxonomy.py, generation of an OTU table with the script make_otu_table.py, summarizing taxa with the script summarize_taxa.py, testing evolutionary distance by building an alignment with the script align_seqs.py, hard sequence filtration of the alignment using a Lane mask with the script filter_alignment.py, generation of a phylogenetic tree with FastTree with the script make_phylogeny.py, rarefaction analyses with the script multiple_rarefactions.py, assessment of alpha diversity with the script alpha_diversity.py, assessment of beta diversity with the script jackknifed_beta_diversity.py, and distance statistics were created with the script dissimilarity_mtx_stats.py. All scripts referenced above are available on the QIIME website and are also available upon request.

### Statistical Analyses

The R Software Package (R Foundation for Statistical Computing, Vienna, Austria) and Microsoft Excel were used to perform the statistical analyses of microbiome data. Student’s *t*-test, a one-way analysis of variance and paired *t*-tests were performed for significance determination (statistical significance required a *P-*value < 0.05). Statistical analyses for other data were performed using Microsoft Excel. Microsoft Excel was used to determine standard deviation and statistical significance using two-tailed paired and unpaired *t*-test (statistical significance required a *P-*value < 0.05). Data composed of pre and post treatment samples within the same group (Treatment T_0_ vs. T_8_) was analyzed using paired *t*-test and to compare the effect of treatment on microbiota (Treatment vs. Control at T_8_), an unpaired *t*-test was performed.

## Results

### Identification of the *Bacillus velezensis* Strains Showing the Best *in vivo* Efficacy in a Murine Wound Model in Preventing *Staphylococcus aureus* Infection

The initial animal model consisted of creating four distinct wounds on the dorsal skin of the mouse and pipetting *S. aureus* Xen29 onto each wound, followed by application of *B. velezensis* strains to designated treatment wounds. This procedure proved inconsistent due to mice licking the bacterial suspensions from the wounds (data not shown). This prompted modification of the protocol wherein bacterial cultures were injected subcutaneously into two regions of the dorsal skin. To ensure that treatment was applied in the same site as the infection, *B. velezensis* spores and *S. aureus* Xen29 were co-administered by mixing in equal parts immediately prior to injection. This procedure ensured that the numbers of injected cells remained consistent, and mouse communal grooming habits did not interfere with the results.

In the first disease challenge using co-inoculation subcutaneously, spores of five *B*. *velezensis* (strains AP143, AP191, AP183, AP218, and AB01) were selected for evaluation of their respective ability to inhibit *S. aureus* Xen29 colonization in a mouse cutaneous wound model. Each mouse in the treatment group was simultaneously challenged with *S. aureus* Xen29 in two independent cutaneous wounds on each mouse back. The *S. aureus* Xen29 suspension and/or spores were pipetted onto wounds of two mice per treatment group. The final concentrations of *S. aureus* Xen29 and *B*. *velezensis* spores per wound were approximately 1.0 × 10^7^ CFU respectively. The results in these small number of animals indicated that co-administration of *S. aureus* Xen29 with spores of *B. velezensis* AP183 resulted in the greatest inhibition of *S. aureus* Xen29 growth and bioluminescence as compared to the other *B. velezensis* strains AP191, AP218, AB01, or AP143, with *B. velezensis* AP191 showing a moderate degree of *S. aureus* inhibition (data not shown).

### Evaluating Inhibition of *Staphylococcus aureus* Xen29 Infection by Two *Bacillus velezensis* Strains

**FIGURE 1:**
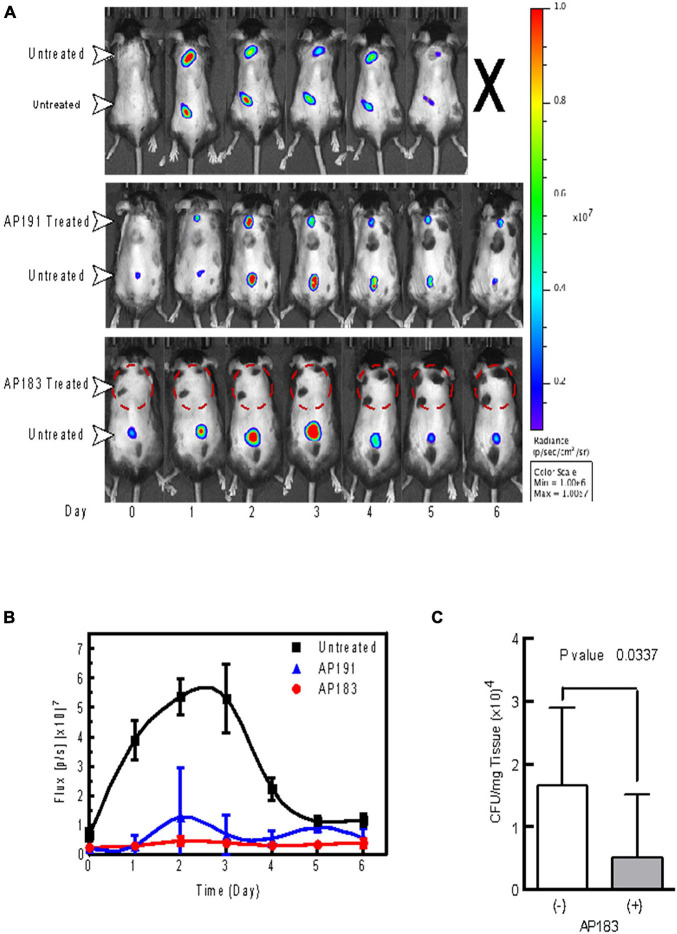
*In vivo* evaluation of *B. velezensis* strains AP183 and AP191 inhibition of *S. aureus* Xen29 in a cutaneous wound model. Mice were given subcutaneous injections of 1 × 10^6^ CFU/mL of *S. aureus* Xen29 between the shoulder blades and on the lower back. **(A)** Upper panel, untreated mouse that succumbed to systemic infection 5 days after initial cutaneous wound challenge. Center panel, mouse treated with *B. velezensis* AP191 culture on upper wound and untreated in the lower wound. Lower panel, mouse treated with *B. velezensis* AP183 culture on upper wound and untreated in the lower wound. **(B)** Bioluminescent flux (photons/second) of *S. aureus* Xen29 in AP183/AP191 treated wounds and untreated controls over time. **(C)** CFU determined for AP183 treated and untreated wounds from excised homogenates plated on TSA.

A subsequent *in vivo* challenge of mice (*n* = 5 in each treatment and control group) with wounds treated with a *B. velezensis* AP183 culture resulted in a significant decrease (*P* < 0.05) of bioluminescence from *S. aureus* Xen29 (Figures 1A,B). Furthermore, we observed a significant reduction (*P* < 0.05) in the number of cultured CFUs in homogenates corresponding to AP183 and supernatant treated wounds (Figure 1C). Injection sites treated with *B. velezensis* AP191 spores and supernatant were less consistent, but still resulted in decreased bioluminescent signal overall as compared to control wounds. Bioluminescent signal in untreated control wounds peaked between days 2 and 3 with a substantial decrease in reported signal by the end of the study. However, one control mouse inoculated with *S. aureus* Xen29 but no *Bacillus* spores succumbed to systemic infection 7 days post-inoculation despite a substantial decrease in bioluminescent signal from wound sites, suggesting that in this control mouse that the *S. aureus* Xen29 had spread beyond the wound site several days after it was subcutaneously inoculated. Because *B. velezensis* AP183 was observed to provide the greatest degree of *S. aureus* inhibition compared to that observed for *B. velezensis* AP191 and other strains, and there was greater knowledge concerning the bioactive metabolite (i.e., bacillusin A) expressed by this strain that was responsible for *S. aureus* growth inhibition, subsequent biofilm and *in vivo* experiments in dairy cows focused on this strain.

### Histological Analysis

**FIGURE 2:**
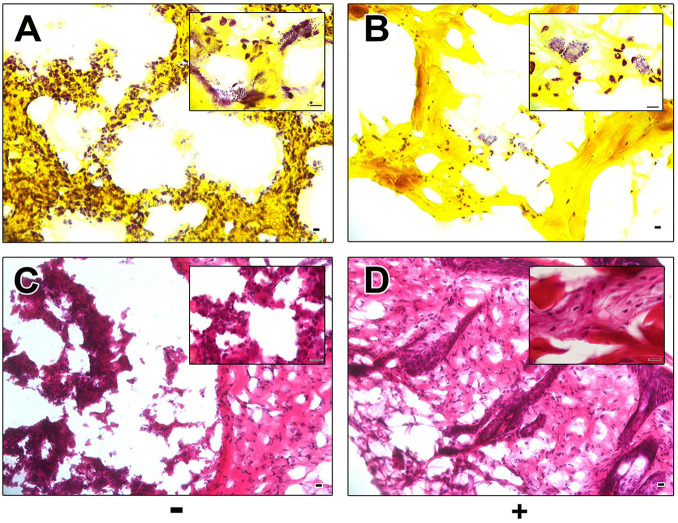
Histological analysis of control and AP183 treated wounds. Gram **(A,B)** and H&E **(C,D)** stained sections of excised wounds infected with *S. aureus* Xen29 in the absence **(A,C)** and presence **(B,D)** of AP183 co-administration. Clusters of Gram-positive cocci were observed in higher frequency in untreated wounds than those treated with *B. velezensis* AP183. Scale indicates 100 μM.

Histological Gram staining revealed an abundance of Gram-positive cocci on sections of excised control wounds as compared to wounds treated with AP183 spores and/or metabolites (Figures 2A,B). However, Gram-positive cocci were also observed in treated wounds. H&E-stained sections of excised tissue appear necrotic and inflamed in contrast to AP183 treated wounds (Figures 2C,D).

### Competition Between *Bacillus velezensis* AP183 and *Staphylococcus aureus* Under *in vitro* Conditions

**FIGURE 3:**
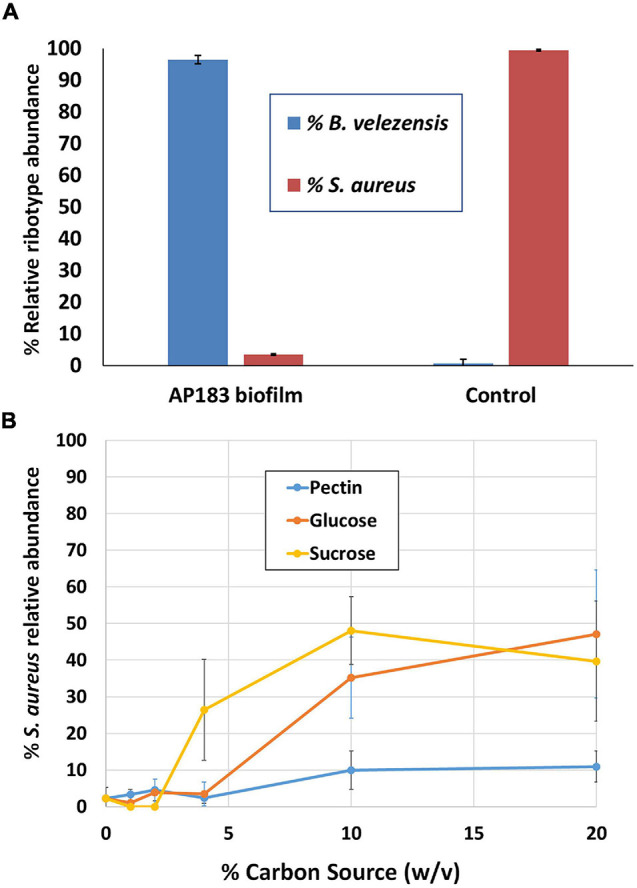
Competition between *B. velezensis* AP183 and *S. aureus* NE1260F when **(A)** the *B. velezensis* AP183 culture forms a biofilm on a tracheostomy tube inner cannula section prior to inoculation with *S. aureus* NE1260F, or **(B)** when *B. velezensis* AP183 and *S. aureus* NE1260F are co-inoculated into M9 minimal medium containing either glucose (can be used by both cultures), sucrose (can be used by both cultures), or pectin (can only be used by *B. velezensis* AP183).

The previous experiments in a mouse wound model indicated that *B. velezensis* AP183 provided the strongest inhibition of *S. aureus* proliferation and systemic infection, among the tested *B. velezensis* strains. In those experiments the *S. aureus* inoculation was performed with a bacterial suspension, and co-inoculated with a *B. velezensis* culture. Because *S. aureus* commonly forms biofilms on medical device substrates that can lead to systemic infections, an experiment was conducted to evaluate if a *B. velezensis* AP183 biofilm formed on a tracheostomy tube inner cannula substrate could impact subsequent *S. aureus* colonization. Due to difficulties in assessing accurate CFU counts from a biofilm, DNA was isolated from biofilm samples in which *B. velezensis* AP183 had formed a biofilm prior to inoculation with *S. aureus* NE1260F or from biofilm formed by *S. aureus* NE1260F only. In the treatment group that was inoculated previously with *B. velezensis* AP183, a significant decrease was observed in *S. aureus* relative abundance on the inner cannula sections (*P* = 0.01) compared to control samples. In the treatment group the *S. aureus* relative abundance was 3.5%, whereas the relative abundance of *Bacillus* was 96.5% (Figure 3A). In contrast, the control group was dominated by 99.4% *S. aureus* (Figure 3A).

The previous experiment showed that a *B. velezensis* AP183 biofilm was able to resist colonization by *S. aureus*. A subsequent experiment was designed to evaluate the relative abundance of these two bacteria when co-inoculated into M9 minimal medium containing different carbon sources. Whereas both *B. velezensis* AP183 and *S. aureus* can grow in the presence of glucose or sucrose, only *B. velezensis* AP183 can use pectin as a sole carbon source. When these two cultures were inoculated into 96-well plates containing each of these carbon sources, it was observed that the relative abundance of *S. aureus* NE1260F increased when the concentration of glucose or sucrose reached very high levels, but no corresponding increase in *S. aureus* NE1260F relative abundance was observed when pectin was the sole carbon source and this difference was significant at a 10% (w/v) concentration of these carbon sources (Figure 3B; *P* < 0.05). While no significant difference was observed when lactose was included as a sugar (*P* = 0.09) the mean relative abundance of *S. aureus* NE1260F in the presence of lactose remained less than 25% even at the highest lactose concentrations (Figure 3B). This experiment revealed that the competition between these bacteria is influenced by the available growth substrate. Subsequent experiments were conducted to evaluate the ability of *B. velezensis* AP183 to antagonize a *S. aureus* biofilm that was formed within a microfluidic device, which similarly showed a strong inhibitory effect of *B. velezensis* AP183 on *S. aureus* viability and resulted in biofilm disruption (data not shown, manuscript in preparation). Collectively, these experiments indicated that *B. velezensis* AP183 can compete with *S. aureus* under different environmental conditions, including in microbial biofilms on a medically relevant substrate, and that this competition is influenced by the growth substrate.

### Antibacterial Activity of *Bacillus velezensis* AP183 Against Mastitis Isolates

The antibacterial activity of *B. velezensis* AP183 against mastitis-inducing pathogens was evaluated by recording the respective zones of inhibition against each isolate. *B. velezensis* AP183 was observed to produce antibacterial metabolites that inhibited the growth of all eight *S. aureus* mastitis isolates, one *S. uberis* mastitis isolate, and one *B. cereus* mastitis isolate (Supplementary Table 1). No inhibitory effects were observed for *B. velezensis* AP183 against a Gram-negative bacterial mastitis isolate, *K. pneumoniae* (Supplementary Table 1).

### *Bacillus velezensis* AP183 at a High Dose Induces a Short-Lived Mammary Inflammatory Response

**FIGURE 4:**
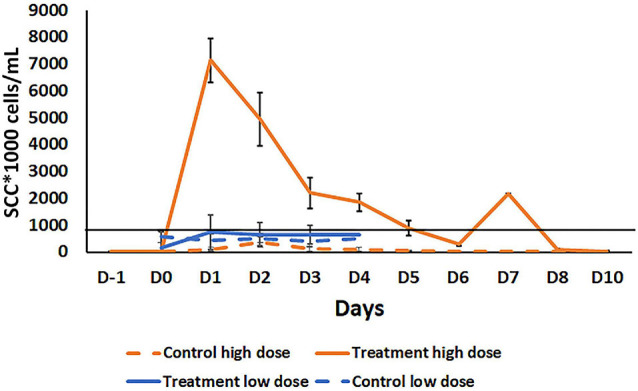
Comparison of somatic cell count (SCC) in milk samples after intramammary inoculation of bovine with either a low (10^3^ CFU) or high (10^9^ CFU) dose of *B. velezensis* AP183. The black line across the graph indicates the bulk-tank SCC threshold that is 750,000 according to USDA. Treatment high dose: Treatment with a high dose (10^9^ CFU) B. *velezensis* AP183; Control high dose: No treatment in high dose group; Treatment low dose: Treatment with low dose (10^3^ CFU) of *B. velezensis* AP183; Control low dose: No treatment in a low dose group. Milk samples in high dose study were collected from pre inoculation day (D-i) to post inoculation days D_o_ to D_10_. In the low dose study, milk samples were collected from D_o_ to D_4_. Though the low dose and high dose inoculation were two different experiments with different animals at different times, all other procedures were the same. Significant differences were found between the low dose and high dose treatment groups in SCC count in milk samples collected from D_o_ to D_4_ (Di, *P* = 6.8 × 10^– 6^; D_2_, *P* = 0.0005; D_3_, *P* = 0.04; D_4_, *P* = 0.03).

To evaluate the ability of *B. velezensis* AP183 to induce an immune response within mammary tissue, and the timing for inflammation resolution, a high dose (approximately 10^9^ CFU) of *B. velezensis* AP183 was inoculated intramammary into healthy bovine mammary glands to evaluate the *in vivo* effects compared to healthy quarters that served as a negative control. This high-dose inoculum induced a significant increase in SCC (approximately 7 × 10^6^ cells/mL) by 24 h post-inoculation which was significantly higher than the SCC observed for the negative control quarters (at 24 h, *P* = 1.5 × 10^–6^; *P* < 0.01 for all post-inoculation time points) (Figure 4). All cows developed localized clinical mastitis with visible abnormal mammary secretions from inoculated quarters without any signs of systemic disease. All quarters infused with the challenge dose of *B. velezensis* AP183 showed visibly evident caseous material in the milk. The California mastitis test indicated an increase in somatic cells for the *Bacillus-*infused quarters and for some non-infused quarters. No signs of systemic infection of elevated temperature were noted in any of the infused cows. All clinical cases resolved within 5 days without antimicrobial treatment and CMT results regressed without any further observation of mastitis symptoms. The somatic cell counts decreased to a normal level (<1.42 × 10^3^ cells/mL) by 10 days post-inoculation. This result as well as results from previous studies (Klostermann et al., 2008; Frola et al., 2012) suggest that, while *B. velezensis* could induce significant inflammation, the inflammatory response resolved within a short period of time. This also suggested that inoculation of mammary tissue with a lower dose of *B. velezensis* AP183 would be less inflammatory. Further analysis of the milk obtained from this preliminary experiment indicated that milk from quarters receiving a high inoculum dose had greater protein and fat content compared with control quarters at all time points with significant differences observed at D_2_ and D_6_ for fat (*P* < 0.05 at both time points) and D_2_ and D5 for protein (*P* < 0.05 at both time points). Interestingly, the lactose and SNF values were less in the treatment group with significant differences observed for lactose levels at Days 1 through 8 (*P* < 0.05 for each time point) and for SNF levels at D_1_, D_4_, and D_5_ (*P* < 0.05 at each time point) (Supplementary Figure 1). The reduced lactose and SNF levels in the treatment group with high SCC in this study is in accordance with the study of dos Reis et al. (2013) but the high protein and fat content of milk from inoculated, high SCC quarters that were observed in this study did not agree with observations made in some other studies (Schultz, 1977; dos Reis et al., 2013).

### Bacillusin A Was Not Detected in Milk Samples From the High Inoculum Experiment

The milk samples from quarters treated with the high dosage of *B. velezensis* AP183 at D_5_ did not have detectable bacillusin A based on LC-MS analysis. The detection of purified bacillusin A indicated that the limit of detection sensitivity was 3 ppm. A possible reason for the poor sensitivity for bacillusin A detection could include its instability (Ravu et al., 2015), or possibly during extraction from milk samples the bacillusin A was degraded or inefficiently recovered. It is unknown whether *B. velezensis* AP183 germinates and expresses bacillusin A within mammary tissues. Further research to provide better recovery of intact bacillusin A from milk samples and to assess bacillusin A biosynthetic gene cluster expression would be helpful in assessing any antibiotic residues present in milk, especially when a lower spore inoculum is used.

### Intramammary Inoculation of *B. velezensis* AP183 at a Low Dose Does Not Induce Clinical Mastitis

A subsequent experiment was conducted to determine the effect of a lower dosage of *B. velezensis* AP183 on the mammary inflammatory response. A lower dose inoculum of 10^3^ CFU *B. velezensis* AP183 did not induce clinical mastitis in any of the inoculated cows. Milk sampled from *B. velezensis* AP183-inoculated quarters was visibly abnormal, and a slight increase of SCC was observed for those samples, although the difference was not significant. In this experiment, the mean SSC was higher in treatment group compared to the control from day D_1_ to D_4_; however, the SCC for milk from *B. velezensis* AP183-inoculated quarters was below the regulatory threshold of bulk-tank SCC which is 750,000 in the United States (Supplementary Figure 2A). The *Bacillus* CFU counts observed from inoculated quarters increased slightly at D_2_ compared to control (Supplementary Figure 2B). In addition, protein concentration was increased in milk from inoculated quarters, while lactose concentration was reduced in the treatment group compared to control. No changes were observed in fat in the treatment group compared to control, though SNF showed a different pattern with a reduction of SNF observed in the treatment group at D_1_ while it increased the control group (Supplementary Figure 3). The significance of this brief change in SNF at D_1_ in terms of milk quality is unknown. While the experiments evaluating the mammary inflammatory response at the two dosages of *B. velezensis* AP183 were performed with different animals, at different times and with different sample sizes, the procedures for inoculation, milk collection, testing and analyzing were the same for these experiments. A comparison of the mammary tissue inflammatory response at low vs. high dose of *B. velezensis* AP183 over the time course post-inoculation revealed significant differences in SCC between low dose and high dose at each treatment day from D_1_ through D_4_ (D_1_, *P* = 6.8 × 10^–6^; D_2_, *P* = 0.0005; D_3_, *P* = 0.04; D_4_, *P* = 0.03), in each case a vigorous inflammatory response was observed for the high dosage of *B. velezensis* AP183 whereas the low dosage never exceeded the regulatory threshold of bulk-tank SCC in the United States (Figure 4).

### Low Doses of *Bacillus velezensis* AP183 Reduces *Staphylococcus aureus* Abundance

**FIGURE 5:**
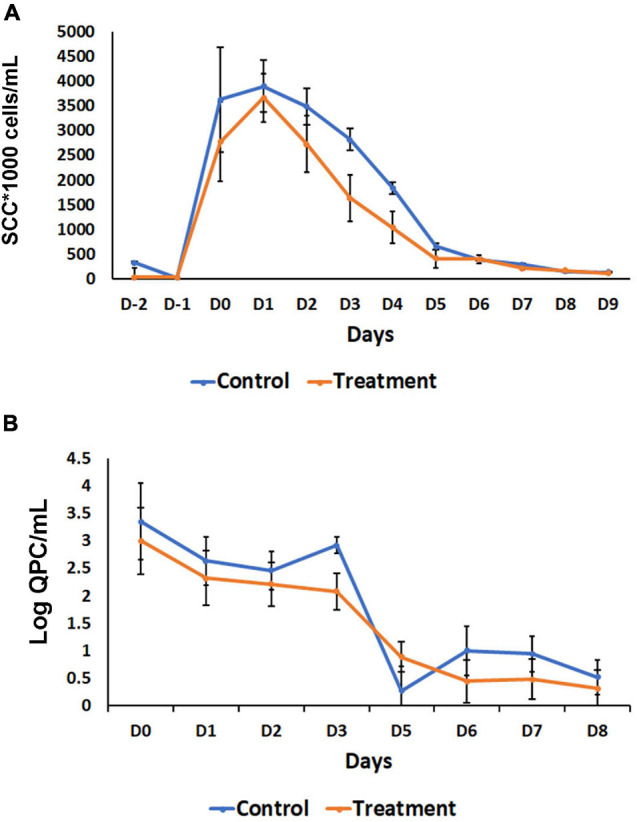
Effect of probiotic treatment on somatic cell counts (SCC) and *Staphylococcus aureus* abundance. **(A)** SCC counts decrease after day one in *B. velezensis* AP183 treated group, when compared with its corresponding control, then both groups return to baseline levels after day five. **(B)** The relative count of *S. aureus* decreased steadily in treatment groups, when compared with the control. The data were expressed as mean ± standard error of log of quantitative plate counts (QPC).

Based on the observation of a significant inflammatory response observed when *B. velezensis* AP183 was inoculated by intramammary infusion at a high dose, we sought to evaluate the ability of this strain to antagonize *S. aureus* within mammary tissues when inoculated at a lower dosage, post-induction of clinical mastitis. All quarters challenged with 10^3^ CFU of *S. aureus* RF122 were observed to have increased SCC with clinical mastitis by 24 h post-inoculation, with decreasing SCC observed at later time points (Figure 5A). Interestingly, after D_1_ a greater SCC reduction was observed in the *B. velezensis* AP183 treatment group as compared to control (Figure 5A), indicating that the inoculated *B. velezensis* AP183 reduced mastitis severity. At 7 days post-inoculation with *B. velezensis* AP183, SCC levels were decreased to approximately day 0 levels. Furthermore, at D_3_ *S. aureus* counts were significantly higher (*P* = 0.04) in control samples as compared to samples treated with *B. velezensis* AP183 (Figure 5B). The levels of fat, protein, lactose and milk solids-not-fat (MSNF) were similar between the control and treatment groups in this lower dose inoculum experiment (Supplementary Figure 4), with no significant differences observed between control and *B. velezensis* AP183-treated quarters (*P* > 0.05).

### *Bacillus velezensis* AP183 at a Low Dose Induces Shifts in the Milk Microbiome When Inoculated

**FIGURE 6:**
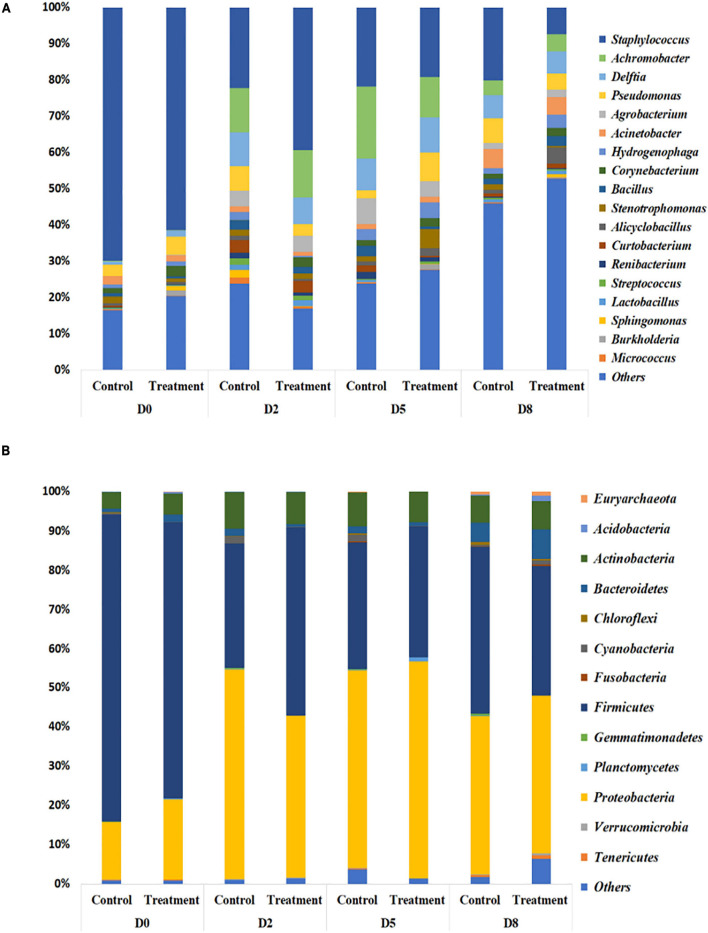
Relative abundance of bacteria in milk from cows with clinical mastitis at the genus level **(A)** and the phylum level **(B)**, with or without *B. velezensis* AP183 probiotic treatment, based on 16S rRNA gene amplicon sequencing. The treatment group corresponds to bovine quarters treated with both *S. aureus* RF122 and *B. velezensis* AP183, whereas the control group corresponds to treatment with *S. aureus* RF122 alone. D_o_, Day before the treatment; D_2_, 2 days post treatment; D_5_, 5 days post treatment; D_5_, 8 days post treatment. The most abundant taxa were sorted by descending order of relative abundance; remaining phylum and genera were grouped as ‘Others.’ Each bar plot of the corresponding category in both phylum and genus level represents the relative abundance of bacteria in each sample.

In the previously described study in which *S. aureus* RF122 was inoculated into all quarters, followed by a low dosage inoculum of *B. velezensis* AP183 into half of these quarters, the milk bacterial community composition was observed to have differences between the control (only *S aureus* RF122) and treatment (*S. aureus* RF122 and *B. velezensis* AP183) groups. The microbiota of milk from cows affected by mastitis was comprised mainly of four bacterial phyla, including taxa affiliated with the Firmicutes, Proteobacteria, Actinobacteria, and Bacteroidetes (Figure 6A). At D_0_, the milk microbiota was dominated by members of the Firmicutes (mean relative abundance of 74.3% at D_0_) followed by Proteobacteria taxa (mean relative abundance of 17.5% at D_0_). Interestingly, the quarters treated with *B. velezensis* AP183 were observed to have a decreased Firmicutes relative abundance over time post-inoculation, changing from 70.4% at D_0_ to 48.0% at D_2_ to 33.1% at D_8_ (Figure 6A). A similar trend for Firmicutes relative abundance was observed for the control group which was observed to be 78.3% at D_0_ to 31.9% at D_2_ to 42.6% at D_8_ (Figure 6A). It is important to note that the increased Firmicutes relative abundance in the control group at the final time point D_8_ was due at least in part to the higher relative abundance of the genus *Staphylococcus*. In contrast, the relative abundance of Proteobacteria taxa were observed to increase in both the control and *B. velezensis* AP183-treated samples at these same time points, with observations of Proteobacteria abundance for control samples changing from 14.6% at D_0_ to 53.4% at D_2_ to 40.1% at D_8_, and similarly the Proteobacteria relative abundance for the *B. velezensis* AP183 treated samples were at 20.4% at D_0_ and increased to 41.1% at D_2_ to 40.1% at D_8_ at the final time point (Figure 6A). The relative abundance of Actinobacteria and Bacteroidetes taxa were observed to be at comparable levels in control and treatment groups.

In addition to phylum-level differences, differences in the relative abundance of bacterial genera were also observed in milk microbiome samples. Milk samples were dominated by *Staphylococcus, Achromobacter*, *Delftia, Pseudomonas, Agrobacterium, Acinetobacter, Hydrogenophaga, Corynebacterium*, and *Bacillus* spp. (Figure 6B). In the quarters treated with *B. velezensis* AP183, a significant decrease was observed in the relative abundance of the genus *Staphylococcus* in milk samples over time (D_0_ vs. D_8_, *P* = 0.003; D_2_ vs. D_8_, *P* = 0.030), with the treatment group showing *Staphylococcus* relative abundance changing from 61.4% at D_0_ to 39.3% at D_2_, and to 7.4% at D_8_. In contrast, in the control group the *Staphylococcus* relative abundance also decreased significantly over time (*P* = 0.002) from 69.8% at D_0_ to 20.2% at D_8_, but no significant difference was observed from 22.3% at D_2_ to 20.2% at D_8_. Most importantly, the results indicated that *B. velezensis* AP183 treatment had a significant effect in decreasing *Staphylococcus* relative abundance relative to the control group at D_8_ (*P* = 0.021). Further, the relative abundance of the genus *Bacillus* was higher in the treatment group (2.9%) at D_8_ compared to control group (1.5%) as would be expected, although this result was not significant. Besides *Staphylococcus*, the only other bacterial genus in which the relative abundance exhibited a significant difference between early time points (D_0_ or D_2_) and D_8_ in control and treatment groups was the genus *Hydrogenophaga*, which in control samples went from 2.3% at D_0_ to 1.4% at D_8_, whereas in treatment samples the *Hydrogenophaga* relative abundance was observed to be at 0.4% at D_0_ and was at 3.5% at D_8_.

**FIGURE 7:**
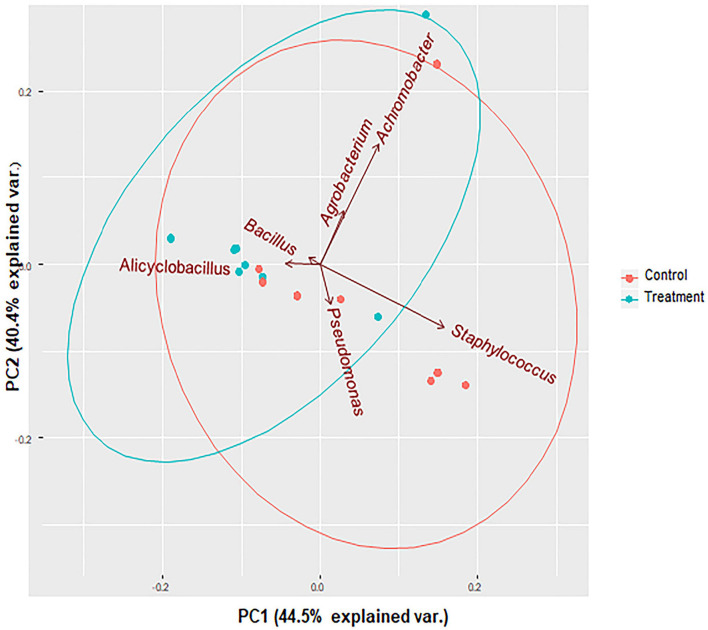
PCA bi-plots of 16S rRNA-based relative abundance according to treatment group. Microbial community profile using beta diversity represented in biplot using weighted Unifrac distances for PCoA analysis. Colors correspond to the control group (red) in which animals were inoculated with only *S. aureus* RF122 and the treatment group (blue) in which animals were inoculated with both *S. aureus* RF122 and *B. velezensis* AP183. PCA indicates distinct differences between the two groups.

Shannon and Simpson diversity indices were not different between the control and treatment groups, but the sample diversity was slightly higher at day 12 in treatment group based on species richness and chao 1 (Table 2), although no statistically significant differences were observed (*P* > 0.05). Interestingly, there were distinct differences between the control and treatment groups observed in the principal component analysis based on weighted Unifrac distances (Figure 7). The treatment group clustered together except for two outliers. While the distribution of data in the control group was dispersed and there were apparent differences from the treatment samples. Principal component 1 explained a large percentage of the variation (86%) for *Staphylococcus* spp. that demonstrated a high correlation of the relative abundance of the *Staphylococcus* with the first principal component.

**TABLE 2:** Comparison of α diversity between treatment and control groups at different time points.

**Day**	**Group**	**Species Richness**	**chao1**	**Shannon**	**Simpson**
D-0	Control	13.3 (±8.9^*^)	35.4 (±27.7)	3.8 (±0.1)	0.97 (±0.0)
	Treatment	14.9 (±9.3)	41.2 (±35.3)	3.8 (±0.1)	0.97 (±0.0)
D-2	Control	17.8 (±5.6)	34.7 (±15.4)	3.9 (±0.0)	0.97 (±0.0)
	Treatment	15.8 (±5.9)	32.7 (±13.7)	3.9 (±0.0)	0.97 (±0.0)
D-5	Control	16.3 (±7.5)	33.3 (±15.4)	3.9 (±0.0)	0.97 (±0.0)
	Treatment	17.3 (±5.8)	30.0 (±10.0)	3.9 (±0.0)	0.97 (±0.0)
D-8	Control	22.0 (±8.0)	37.3 (±12.4)	3.9 (±0.0)	0.97 (±0.0)
	Treatment	24.9 (±9.7)	44.3 (±16.5)	3.9 (±0.0)	0.97 (±0.0)

*Results are expressed as mean values ± standard deviation.

## Discussion

The primary objective of this study was to identify specific probiotic bacteria with the capacity to inhibit *S. aureus* colonization under various conditions that are relevant to human and veterinary medicine. The key considerations used for evaluating candidate probiotic bacteria were safety, efficacy, and commercial potential (i.e., shelf-life). For these reasons, our efforts focused on identifying endospore-forming members of the genus *Bacillus* that have efficacy in preventing *S. aureus* infections, without any known potential for pathogenicity. These criteria narrowed our search to *B. velezensis* strains available in an Auburn University microbial culture collection that had shown the ability to produce bioactive metabolite(s) that inhibit MRSA growth. However, none of these strains had been evaluated under *in vivo* conditions for *S. aureus* antagonism. The preliminary studies using a mouse wound model revealed that *B. velezensis* AP183 was the most effective strain at inhibiting *S. aureus* growth and bioluminescence and was the most promising candidate for future investigation. The antibacterial activity of AP183 is presumed to be due primarily to the production of secondary metabolites, in particular the macrodiolide antibiotic bacillusin A that was previously shown to exhibit strong *in vitro* bactericidal activity against MRSA and vancomycin-resistant enterocci (Ravu et al., 2015). While bacillusin A was found to be a potent inhibitor of several relevant pathogens, it was determined to be chemically unstable in organic solvents and prone to photoisomerization as indicated by LC-MS analysis (Ravu et al., 2015). The photoisomerization and chemical instability in organic solvents ultimately resulted in a low isolation yield of the compound, thereby making it unlikely that purified bacillusin A would be a viable candidate for use as an oral or injectable antibiotic in the clinical setting. However, the biochemical instability of this compound might be advantageous for topical treatment of infected wounds where rapid elimination of antibiotic residues may be preferred. For instance, prophylactic topical application on dressings for severe burn wounds, wherein exposed tissues arereadily infected by *S. aureus* and other pathogens, often resulting in patient mortality (Avni et al., 2010).

The observation that *B. velezensis* AP183 provided the best inhibition of *S. aureus* proliferation and bioluminescence in a mouse wound model could be due to a combination of factors, including the production of secondary metabolites, competitive inhibition for growth substrates and/or other mechanisms. Even in the absence of *B. velezensis* AP183, bioluminescent signal intensity was consistently observed to decrease in control wounds beginning around day four of infection. This decrease in bioluminescent signal may be due to many factors, but histological analysis suggests that dissemination to distant sites from systemic infection may have resulted in some cases. Gram-stained sections of a heart of one control mouse that succumbed to systemic infection revealed clusters of Gram-positive cocci in the aortic region of the heart (data not shown). This observation is consistent with the ability of *S. aureus* to disseminate from a cutaneous wound and establish life-threatening systemic infections at distant sites. In the presence of *B. velezensis* AP183 and its metabolites, the *S. aureus*-derived bioluminescence was almost completely eradicated even at early time points. In contrast, the same degree of *S. aureus* inhibition within a mouse wound was not observed for *B. velezensis* AP191, which did show the capacity for *S. aureus* growth inhibition under *in vitro* conditions. The difference in *in vivo* efficacy between these two *B. velezensis* strains may be due to differences in the secondary metabolites expressed. The *S. aureus* inhibitory activity of *B. velezensis* AP191 observed under *in vitro* conditions has not been attributed to a specific metabolite, nor does this strain have the encoded capacity to produce bacillusin A (data not shown). Based on the data available from the mouse wound experiments and the previously described structure and function of bacillusin A, *B. velezensis* AP183 was selected for all subsequent experiments.

The competitive interactions between *B. velezensis* AP183 and *S. aureus* are expected to be influenced by many different factors. The expression and stability of bacillusin A in association with host tissues is hypothesized to be a key factor in this interaction, and future studies examining the role of bacillusin A in mediating *S. aureus* control *in vivo* will be important in our understanding of these interactions and the degree to which secondary metabolites are involved, relative to other mechanisms such as competitive exclusion and substrate utilization. A newly constructed genetic mutant of *B. velezensis* AP183 that lacks the biosynthetic gene cluster responsible for bacillusin A will be especially useful in evaluating these molecular interactions (manuscript in preparation). In addition to metabolite-mediated inhibition of *S. aureus*, the role of biofilm formation in these bacterial interactions was explored in this study. By first allowing *B. velezensis* AP183 to grow as a “pioneer organism” that formed a biofilm on sections of a tracheostomy tube inner cannula, these biofilm-colonized surfaces significantly reduced *S. aureus* populations based on ribotype relative abundance data. Considering that *S. aureus* commonly forms biofilms on medical implants that can result in life-threatening infections (Oliveira et al., 2018), there could be prophylactic value to establishing a beneficial bacterial biofilm that would be recalcitrant to biofilm formation by opportunistic pathogens. The growth substrate available to these bacteria also appears to impact their competitive interactions, as a significant reduction in *S. aureus* relative abundance was also observed when co-inoculated with *B. velezensis* AP183 in the presence of pectin, as opposed to other sugars such as glucose or sucrose that both cultures could readily utilize as a carbon and energy source. In association with host tissues, there will certainly be a diversity of growth substrates and interactions with host cells, that contribute to the complex molecular interactions that impact microbial physiology and pathogenesis. Given this complexity, there is no substitute for evaluating these interactions under *in vivo* conditions, and it was important to evaluate the ability of *B. velezensis* AP183 to impact *S. aureus* proliferation and infection in an animal disease model of significant agricultural relevance.

This is the first study in which a *B. velezensis* isolate has been evaluated for the ability to prevent or to treat bovine mastitis. The *in vivo* efficacy observed for *B. velezensis* AP183 in inhibiting *S. aureus* in a mouse wound model, and the strong *in vitro* growth inhibitory activity observed for *B. velezensis* against all the mastitis-derived *S. aureus* isolates tested in this study suggested that *B. velezensis* AP183 may have the ability to inhibit a broad spectrum of Gram-positive bacterial taxa that can induce mastitis. Given that a complete *B. velezensis* AP183 genome sequence is available (Nasrin et al., 2015), and this strain is predicted to also encode the biosynthetic gene cluster for the antibiotic difficidin that has been shown to inhibit Gram-negative pathogens such as *Aeromonas hydrophila* and *Xanthomonas campestris* pv. vesicatoria (Hossain et al., 2015), we subsequently evaluated the activity of *B. velezensis* AP183 against *A. hydrophila* ML09-119 (Tekedar et al., 2013). While this strain did not inhibit the growth of *K. pneumoniae*, we did observe growth inhibitory activity expressed by *B. velezensis* AP183 against *A. hydrophila* ML09-119 (data not shown), suggesting that difficidin and/or other metabolites produced by this strain are active against some Gram-negative bacteria as well. Collectively, these results indicate that *B. velezensis* AP183 actively produces bioactive secondary metabolites (i.e., bacillusin A, difficidin) that are active against diverse bacterial mastitis isolates and that *B. velezensis* AP183 should be further evaluated for its ability to reduce clinical mastitis.

Prior to evaluating interactions between *B. velezensis* AP183 and *S. aureus* in mammary tissues, it was important to determine the mammary tissue immune response to inoculation with *B. velezensis* AP183. We observed that intramammary inoculation of *B. velezensis* AP183 at a high dosage induced a short-term increase of SCC with clinical mastitis that rapidly resolved by five days post-inoculation. The results obtained in this study are like the study of Mignacca et al. (2017), who observed a short-term increase in SCC after probiotic *L. lactis* subsp. Lactis LMG 7930 treatment and rapid decline of SCC to pre-treatment level 7 days post inoculation. Further, Crispie et al. (2008) observed increased numbers of polymorphonuclear leucocytes at 2 days post-inoculation that decreased by 5 days post-inoculation. We also observed that low dose *B. velezensis* AP183 caused low grade sub clinical mastitis that increased SCC but below the threshold level of bulk tank SCC level for US. Importantly, when the lower dose of *B. velezensis* AP183 was administered after *S. aureus*-induced clinical mastitis, a significant decrease in *S. aureus* was observed by D_3_ in the treatment group relative to the control group, and these results were also shown by decreased relative abundance of *Staphylococcus* from the 16S rRNA gene amplicon sequencing results as well. Collectively, these results demonstrated that the SCC and *Staphylococcus* count reduction was more rapid in the bovine quarters exposed to *B. velezensis* AP183, indicating that *B. velezensis* AP183 had some efficacy in reducing the severity of staphylococcal-induced mastitis.

Our data showed that the most abundant bacteria detected in milk from bovine mastitis quarters were from the phyla Proteobacteria, Firmicutes, Actinobacteria, and Bacteroides, which were similar observations to previous studies (Lima et al., 2017; Catozzi et al., 2019; Taponen et al., 2019). Furthermore, a lower relative abundance was observed for bacterial taxa affiliated with the phyla Verrucomicrobia, Cyanobacteria, and Acidobacteria, which corresponds well with the results obtained from the study of Catozzi et al. (2019). In this study, differences were found primarily in the relative abundances of bacterial taxa affiliated with the phyla Firmicutes and Proteobacteria in milk between the treatment and control groups.

The differences of the relative abundance of the top microbiota of milk between treatment and control at D_8_ were observed in *Staphylococcus, Bacillus*, and *Alicyclobacillus* taxa. The major finding of this study was the *Staphylococcus* relative abundance was significantly decreased in the treatment group relative to the control group at D_8_, indicating that *B. velezensis* AP183 inoculation was effective in reducing the relative abundance of *S. aureus* in mastitis quarters. Interestingly, the relative abundance of *Bacillus* was slightly higher in the treatment group at D_8_ in this study which supports a negative correlation between the relative abundance of *Bacillus* and *Staphylococcus*. The only other genus in which a significant change was observed in the milk microbiome was an increase in the relative abundance of the genus *Alicyclobacillus* from D_0_ to D_8_ in the treatment group. This soil borne genus is usually associated with the spoilage of fruit juices (Groenewald et al., 2009; Bevilacqua et al., 2015). This may indicate that the growth substrates used by *Alicyclobacillus* spp. are increased in their availability after inoculation with *B. velezensis* AP183 (Laurence et al., 2014; Salter et al., 2014; Weiss et al., 2014).

## Conclusion

In conclusion for the bovine mastitis experiments, this study observed that intramammary inoculation of *B. velezensis* AP183 at a relatively low dose in a bovine model of *S. aureus*-induced mastitis reduced the relative abundance of *Staphylococcus* and had minor effects on the rest of the milk-associated microbiota. Despite the small number of cows available for use in this study, significant differences were observed that support additional research to better understand how the use of a beneficial microorganism can antagonize the bacteria such as *S. aureus* that can induce chronic mastitis. Future studies can contribute to understanding the molecular interactions that impact *Bacillus* and *Staphylococcus* interactions with each other, the host immune response and the ways in which an understanding of these interactions can best be applied to reduce the prevalence of mastitis in dairy animals, and in other applications in human and veterinary medicine.

## Importance

*Staphylococcus aureus*, an opportunistic pathogen that is commonly resistant to antibiotic treatment, is responsible for potentially life-threatening infections in human and veterinary medicine. In this study, we present for the first-time *in vivo* studies demonstrating the inhibition of *S. aureus* by *B. velezensis* AP183 in a mouse cutaneous wound model, and in a dairy cow mastitis model. Biofilms formed by *B. velezensis* AP183 on a tracheostomy tube inner cannula substrate were resistant to colonization by *S. aureus.* Furthermore, a low dose inoculum of *B. velezensis* AP183 inoculated into bovine mammary tissue did not induce clinical mastitis but did significantly reduce the *Staphylococcus* relative abundance within a milk microbiome. The results of this study provide support for the use of *B. velezensis* AP183 and/or its metabolites as an alternative to traditional antibiotics for the treatment of *S. aureus* infection in humans and animals.

## Data Availability Statement

The datasets presented in this study can be found in online repositories. The names of the repository/repositories and accession number(s) can be found below: https://www.ncbi.nlm.nih.gov/genbank/, accession number CP029296.1.

## Ethics Statement

The animal study was reviewed and approved by Auburn University’s Institutional Animal Care and Use Committee (IACUC).

## Author Contributions

SA, AB, SN, AA, TH, HM, CR-I, ED, and ML conducted the experiments and analyzed the data. HM, MS, ED, PP, CW, and ML designed the experiments and made other intellectual contributions. ED, PP, CW, and ML wrote proposals that provided funding for this research. All authors contributed to the writing and editing of this manuscript.

## Conflict of Interest

The authors declare that the research was conducted in the absence of any commercial or financial relationships that could be construed as a potential conflict of interest.

## Publisher’s Note

All claims expressed in this article are solely those of the authors and do not necessarily represent those of their affiliated organizations, or those of the publisher, the editors and the reviewers. Any product that may be evaluated in this article, or claim that may be made by its manufacturer, is not guaranteed or endorsed by the publisher.
