# Supplementary figures and images for: Bacillus velezensis AP183 Inhibits Staphylococcus aureus Biofilm Formation and Proliferation in Murine and Bovine Disease Models
Source: Front Microbiol. 2021 Oct 8;12:746410. doi: 10.3389/fmicb.2021.746410 (PMC8533455; doi:10.3389/fmicb.2021.746410)

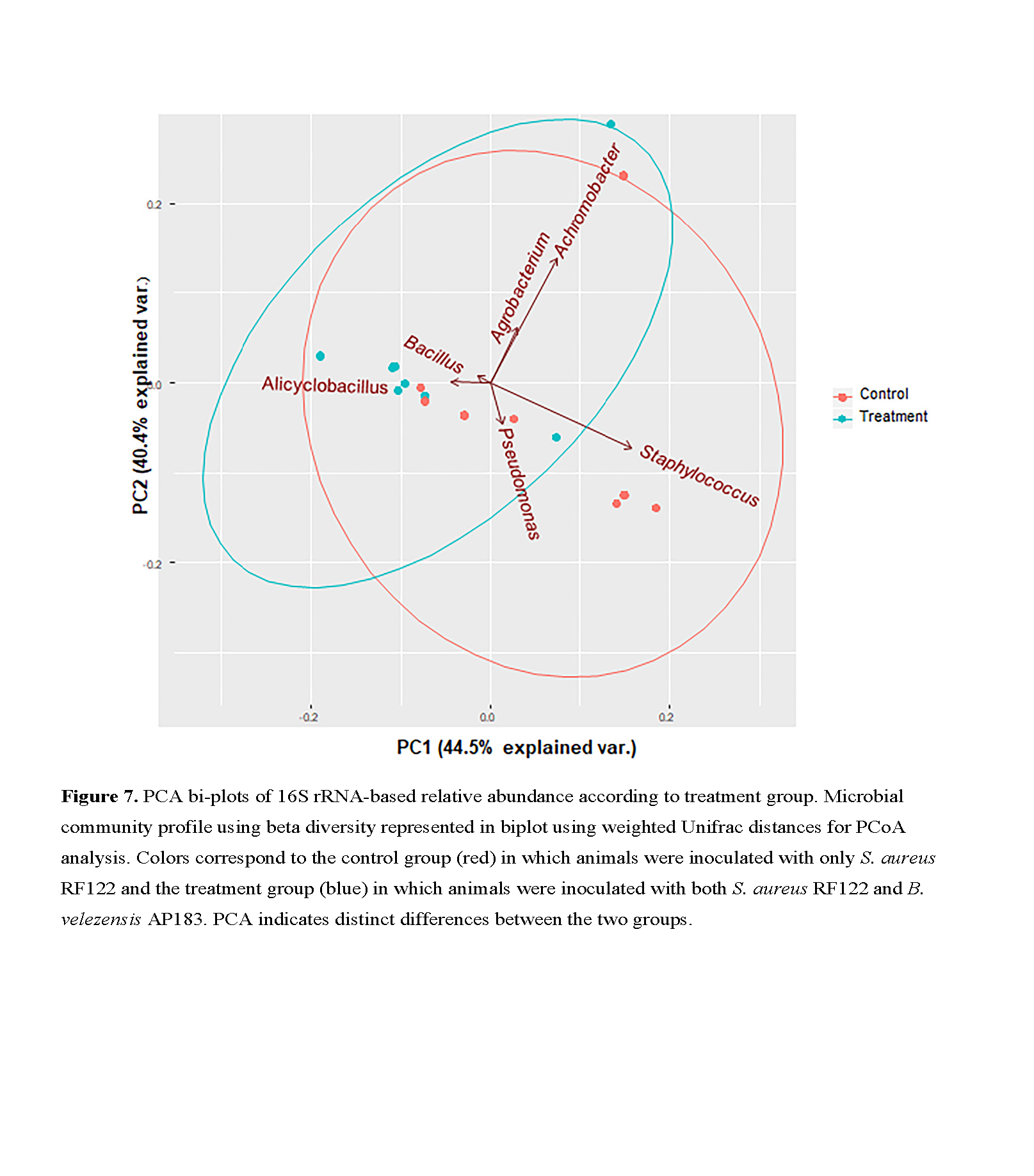

Supplement: Supplementary file 1 [file Image_1.tif]

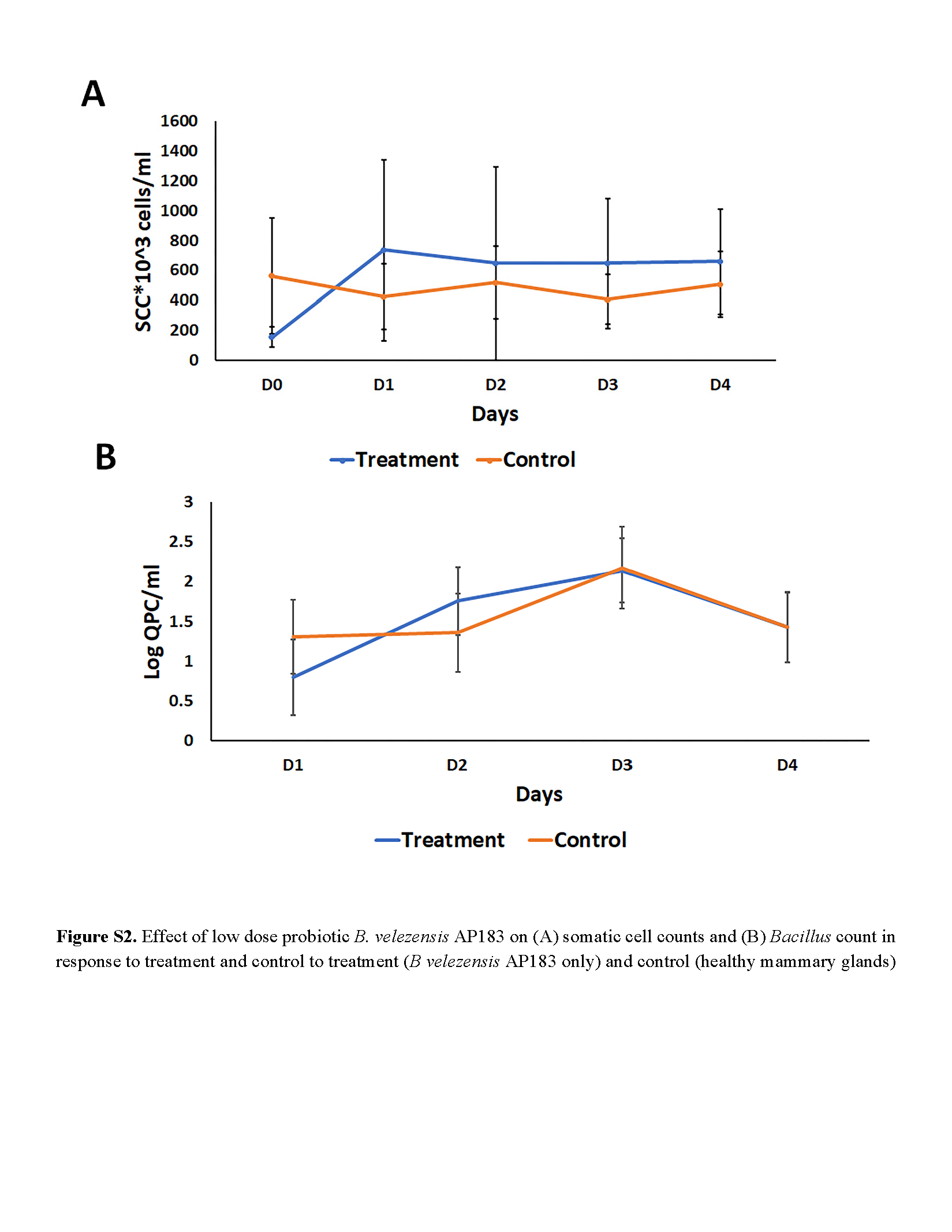

Supplement: Supplementary file 2 [file Image_2.tif]

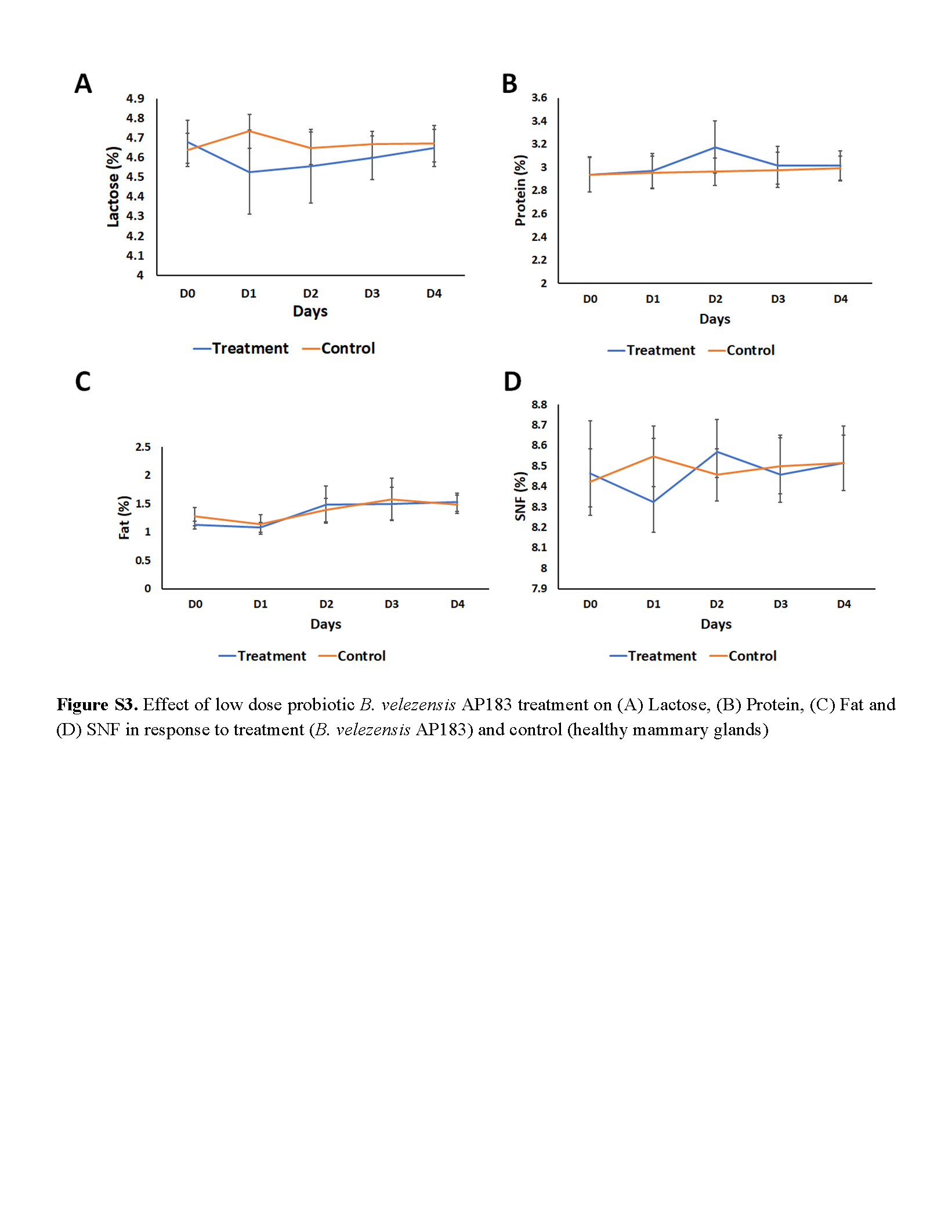

Supplement: Supplementary file 3 [file Image_3.tif]

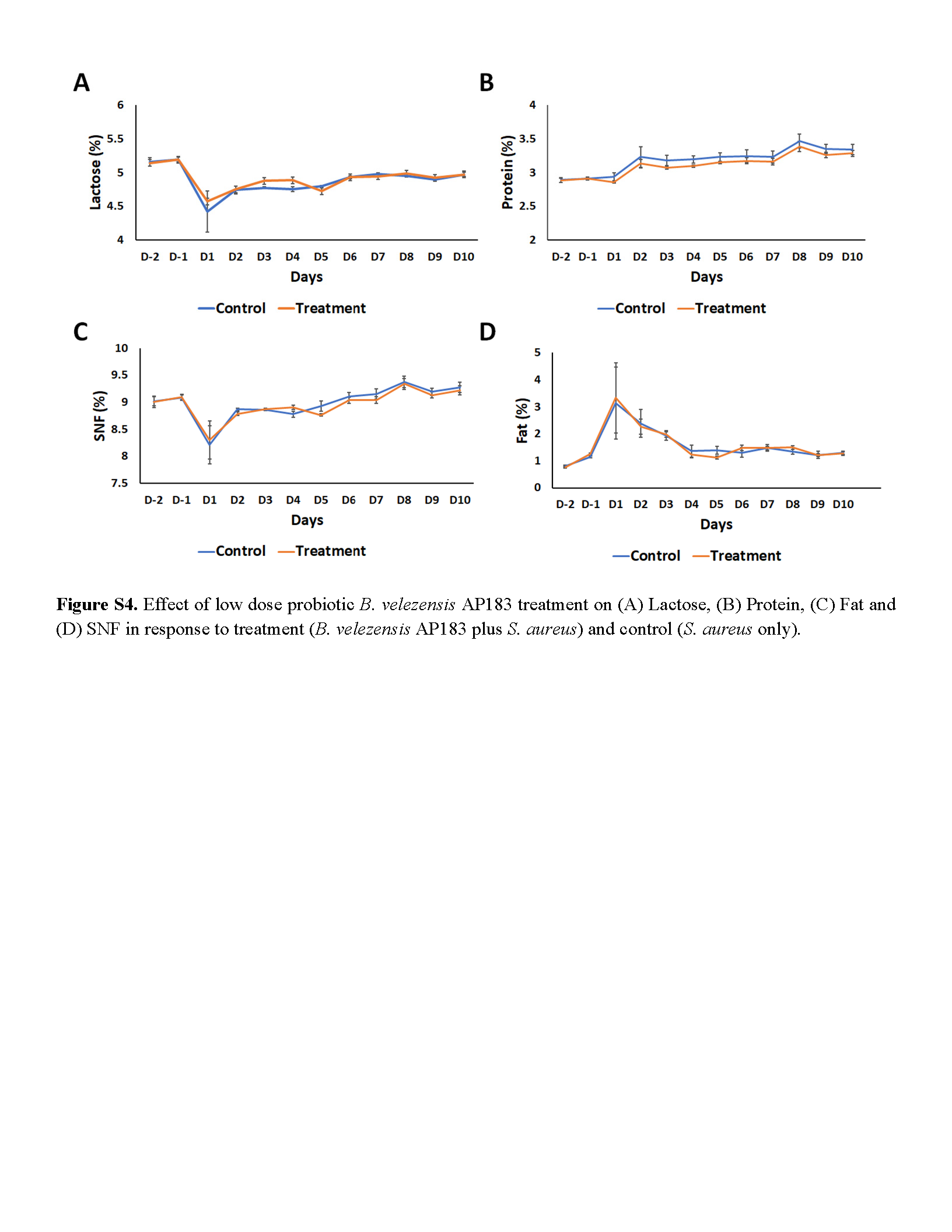

Supplement: Supplementary file 4 [file Image_4.tif]
