# Supplementary material for: Bacillus velezensis AP183 Inhibits Staphylococcus aureus Biofilm Formation and Proliferation in Murine and Bovine Disease Models
Source: Front Microbiol. 2021 Oct 8;12:746410. doi: 10.3389/fmicb.2021.746410 (PMC8533455; doi:10.3389/fmicb.2021.746410)
Supplement: Supplementary file 2 [file Table_1.docx]

Table S1. Antimicrobial activity of *B. velezensis* AP183 against mastitis-causing bacteria.

Organism	Strain	Test result[1]
S. aureus	1171-09	S
S. aureus	4366-13	S
S. aureus	1052-1	S
S. aureus	2226-11	S
S. aureus	1779-09	S
S. aureus	3851-07	S
S. aureus	3651-09	S
S. aureus	RF122	S
S. uberis	22272-08	S
K. pneumoniae	1583-07	R
B. cereus	NRS 1595	SS

[1]S = Susceptible to *B. velezensis* AP183 metabolites, based on a zone of growth inhibition >5 mm; SS = Slightly susceptible to *B. velezensis* AP183 metabolites, based on a zone of growth inhibition <5 mm; R = Resistant to *B. velezensis* AP183 metabolites, no growth inhibition was observed.
